# The cost of perfection: Periodontal risks of the Hollywood smile

**DOI:** 10.34172/japid.4386

**Published:** 2026-09-12

**Authors:** Adileh Shirmohammadi, Sina Ghertasi Oskouei

**Affiliations:** ^1^Dental and Periodontal Research Center, Tabriz University of Medical Sciences, Tabriz, Iran; ^2^Department of Periodontics, Faculty of Dentistry, Tabriz University of Medical Sciences, Tabriz, Iran; ^3^Section of Digital Dentistry, Faculty of Dentistry, Tabriz University of Medical Sciences, Tabriz, Iran

**Keywords:** Dental implants, Dental veneers, Esthetics, Gingival recession, Periodontal attachment loss, Periodontium, Quality of life

 Patient demand for uniformly white, high-value, symmetrically aligned smiles, popularized by social media, has led to a marked increase in elective porcelain laminate and veneer procedures.^1^ While contemporary ceramics and adhesive protocols can improve appearance and oral health-related quality of life when correctly indicated,^2,3^ the periodontal consequences of aggressive preparation, subgingival margin placement, and poorly contoured restorations remain under-appreciated. The decisive clinical questions concern how these restorations interact with the supracrestal tissue attachment, gingival phenotype, and long-term soft tissue architecture, and whether short-term visual perfection justifies risks to periodontal stability and future implant sites.

 The pathway from aesthetic ambition to periodontal injury usually begins with irreversible removal of enamel and dentin. Quantitative studies have shown that even conservative veneer preparations can eliminate a substantial fraction of coronal tooth structure.^4^ When laminates are used to mask crowding or proclination instead of orthodontic alignment, reduction becomes still more extensive.^5,6^ The resulting thin residual walls and often subgingival finish lines set the stage for soft tissue complications. In the pursuit of seamless emergence profiles and ideal tooth display, margins are frequently placed within or apical to the supracrestal tissue attachment. Equally damaging are over-contoured or overhanging margins and bulky cervical anatomy created solely to satisfy white aesthetic ideals. These plaque-retentive surfaces impede hygiene, promote biofilm accumulation, and sustain chronic inflammation even when the initial preparation appears technically acceptable.

 The supracrestal tissue attachment, comprising junctional epithelium and connective tissue attachment, represents a physiologic dimension that the periodontium re-establishes when violated.^7^ Placement of margins inside this zone, compounded by over-contoured restorative contours, triggers chronic inflammation as the tissues attempt to recreate a protective seal. The common sequelae include apical migration of the gingival margin, loss of papilla fill, recession, localized attachment or crestal bone loss, and persistent bleeding or food trapping.^7,8^ These changes are phenotype-dependent. Thin scalloped biotypes with limited keratinized tissue are especially vulnerable to recession and open gingival embrasures, while thick flat biotypes may respond with persistent inflammation and pocketing.^8,9^ When multiple anterior teeth are treated aggressively, the cumulative effect is a fragile mucogingival environment that is difficult to maintain and aesthetically unstable. Long-term clinical data on porcelain veneers document both survival and a spectrum of biological complications, including soft tissue and plaque-related sequelae.^10,11^

 The downstream implant implications are substantial. Progressive soft tissue deficits, altered ridge contours, and chronic inflammatory changes after years of inflamed or receded margins create less favorable conditions for extraction and implant placement.^12‒14^ Pink aesthetic reconstruction becomes more complex, often requiring soft tissue grafting, connective tissue procedures, or staged ridge augmentation that could have been avoided. Thus, an elective aesthetic intervention can convert a relatively straightforward natural dentition into a site requiring advanced periodontal and implant reconstructive therapy.^12‒14^

 Diagnostic discipline must therefore prioritize periodontal parameters. Comprehensive evaluation should include probing depths, clinical attachment levels, gingival phenotype classification, assessment of keratinized tissue width, estimation of the supracrestal tissue attachment via bone sounding or three-dimensional imaging when indicated, and occlusal and functional analysis before any smile design visualization. Contemporary Digital Smile Design and related digital planning concepts increasingly emphasize pink aesthetics alongside white aesthetics, explicitly calling attention to gingival health, symmetry, and tissue stability.^15^ This evolution is welcome and necessary. Yet these tools remain subordinate to biologic feasibility. When used primarily as marketing devices to sell a predetermined ideal, or when pink aesthetic rhetoric is not matched by respect for tissue dimensions and cleansable contours, they risk accelerating iatrogenic pathways rather than preventing them. Case selection must explicitly weigh orthodontic alternatives, additive or no-preparation options, patient hygiene capacity, and the realistic prospect of lifelong maintenance.^6,16^

 Psychosocial drivers amplify the problem. Patients frequently arrive with expectations calibrated to filtered images that disregard individual soft tissue anatomy, lip dynamics, and aging.^1^ After the initial period of satisfaction, many confront altered surface characteristics, the need for repairs or replacements, and the permanent loss of natural tooth structure and gingival architecture.^17^ While aesthetic improvement can enhance self-perception and oral health-related quality of life,^2,3^ short-term satisfaction scores do not capture longer-term soft tissue deterioration or the broader determinants of quality-of-life indices. These determinants can encompass chronic bad odor from inflamed tissues, an inflamed appearance, recurrent bleeding gums, food traps created by over-contoured or overhanging margins, more frequent visits for retreatment or correction, the need for advanced or less predictable procedures arising from poor initial planning or execution, the cumulative costs and time required, and the overall negative perception of personal oral health and aesthetics. Informed consent must therefore address not only porcelain longevity but also risks to the periodontium, potential future implant complexity, and these lasting impacts on quality of life.

 Aesthetic enhancement remains a legitimate therapeutic goal and can confer genuine benefits. That fact does not license an appearance-driven narrative that subordinates biologic boundaries.^5,18^ A hierarchy of intervention grounded in periodontal preservation is required: professional bleaching and microabrasion for discoloration; orthodontic alignment for positional problems; additive composite or true minimal- or no-preparation ceramics when form must be modified; and conventional preparation only when structural or restorative necessity is clear.^16,19^ Restorations, when indicated, must feature cleansable emergence profiles and margins free of overhangs or excessive contour. Success metrics should be both periodontal and quality-of-life oriented: stability of attachment levels, absence of inflammation, maintenance of papilla height and phenotype integrity, soft tissue conditions favorable for any future implants, and sustained freedom from the burdens that erode quality of life, evaluated a decade after treatment rather than on the day of cementation.

 Periodontists, implant surgeons, and restorative colleagues share responsibility for resisting trend-driven enamel sacrifice, violation of the supracrestal tissue attachment, and creation of plaque-accumulating anatomy. Interdisciplinary planning that integrates phenotype-aware risk assessment, respect for biologic dimensions, genuine attention to pink aesthetics, and preservation of future implant sites should be standard.^16^ Educational curricula and clinical guidelines need clearer differentiation between indicated aesthetic rehabilitation and elective homogenization. Priority research questions include long-term soft tissue stability, patient-reported outcomes and quality-of-life indices after extensive veneer therapy, the periodontal impact of restorative contour and margin quality, comparative effects of orthodontic versus restorative camouflage of crowding, phenotype-specific risk stratification, and the real-world influence of digital planning tools on preparation aggressiveness, margin location, and respect for pink aesthetics.

 The most sophisticated aesthetic result is not a standardized high-value smile that photographs well under studio lighting, but a harmonious dentition whose periodontal tissues remain healthy, stable, and reconstructible over time. In the era of social media-driven smile ideals, protecting the periodontium is not a secondary consideration; it is the primary measure of clinical integrity.

## Competing Interests

 At the time of submission and publication, Dr. Shirmohammadi served as the Editor-in-Chief, and Dr. Oskouei served as an Associate Editor of the *Journal of Advanced Periodontology & Implant Dentistry* (JAPID). The authors declare no other competing interests.
